# Supplementary material for: Are carbon emissions trading and green financial instruments synergistic? -Comprehensive quantitative research based on content analysis
Source: PLoS One. 2024 Mar 7;19(3):e0298601. doi: 10.1371/journal.pone.0298601 (PMC10919601; doi:10.1371/journal.pone.0298601)
Supplement: S1 Data — (ZIP) [file pone.0298601.s001.zip › Data/地级市Z_F工作报告.zip!/╣π╢1⁄2/╣π╓▌╩╨/└┤╫╘╣1⁄2╓┌║┼í╛┬φ┐╦╩2╛▌═°í┐_2011.docx]

各位代表：

　　我代表广州市人民政府，向大会作工作报告，请予审议，并请政协委员和其他列席人员提出意见。

　　一、“十一五”时期和2010年工作回顾

　　“十一五”时期，是我市发展史上具有里程碑意义的五年。在党中央、国务院和省委、省政府及市委的正确领导下，我们坚持以科学发展观统领全局，认真贯彻实施《珠江三角洲地区改革发展规划纲要》（以下简称《规划纲要》），积极应对国际金融危机的严峻挑战，克服低温雨雪冰冻等自然灾害的严重影响，全力调结构、转方式、促发展、办亚运、惠民生，“十一五”规划主要任务全面完成，改革开放和现代化建设取得重大成就。

　　过去的五年，是我市经济发展实现历史性跨越，综合实力显著提升的五年。全市经济以年均13.5％的速度快速增长，2010年地区生产总值突破1万亿元，达10604.48亿元，同比增长13％，比“十一五”预期目标增加1105亿元，比2005年翻了一番多。预计人均生产总值超10万元，比“十一五”预期目标增加1万元以上，比2005年增长74％。财政总收入3348亿元，同比增长26％，其中地方一般预算财政收入872.65亿元，同比增长24.2％，分别是2005年的2.23倍和2.35倍。金融机构本外币存贷款余额分别为23953.96亿元和16284.31亿元，均比2005年翻了一番多。

　　过去的五年，是我市集中力量筹办第16届亚运会和首届亚残运会，城市知名度和影响力显著提升的五年。我们成功举办了一届高水平、有特色的亚洲文化体育盛会，实现了“两个亚运同样精彩”的目标，谱写了亚运史上的辉煌篇章，有力促进了广州经济社会发展，市民素质明显提升，城市凝聚力和影响力极大增强。

　　过去的五年，是我市城市功能日臻完善，国家中心城市地位和作用显著提升的五年。《规划纲要》赋予广州国家中心城市地位，广州的发展上升到国家战略层面，空港、海港、铁路港、信息港等现代化重大基础设施建设全面提速。2010年，机场旅客吞吐量突破4000万人次，跻身世界前15名；港口货物吞吐量、集装箱吞吐量分别突破4亿吨和1200万标箱，均居世界前6名；高速公路总里程达537公里，地铁运营总里程达236公里。城市综合服务功能全面提升，发展承载能力大幅增强。

　　过去的五年，是我市环境面貌发生巨大变化，城市形象显著提升的五年。我们以“敢想、会干、为人民”的精神，开展了大规模的城市环境综合整治，广州荣获国家环境保护模范城市、国家卫生城市、国家园林城市、国家森林城市称号，获得世界水资源论坛水环境治理奖第一名和国际可持续交通奖，实现了“天更蓝、水更清、路更畅、房更靓、城更美”的目标，赢得了广大市民群众的普遍认可和国内外来宾的广泛赞誉。

　　过去的五年，是我市进一步解放思想，改革创新和开放合作水平显著提升的五年。通过开展新一轮解放思想大讨论和深入学习实践科学发展观活动，进一步解放和发展了生产力。地方金融机构改革重组和城市建设投融资体制改革取得重大进展，社会管理、文化体制等重点领域改革取得新突破。2010年，市属经营性国有资产总量突破7000亿元，比2005年翻了一番多，民营经济占全市GDP比重达38.25％，外贸进出口总值突破一千亿美元，广州参与国际竞争与合作的能力进一步增强。

　　过去的五年，是我市社会事业全面进步，人民生活水平显著提升的五年。五年来，全市共投入2539.3亿元财政资金用于改善民生和发展社会事业，是“十五”时期的2.35倍；新增就业人数341.6万人；城乡“五险”参保人数和企业退休人员养老金均比2005年翻了一番多。建设保障性住房8.07万套，惠及24万多城镇居民。2010年，城市居民人均可支配收入30658元，农村居民人均纯收入12676元，分别比“十一五”预期目标增加5658元和2876元，比2005年分别增长67.6％和79％。

　　五年来，我们主要做了以下八个方面工作：

　　（一）着力构建以服务经济为主体的现代产业体系。

　　抓住国际金融危机形成的倒逼机制，出台关于建设现代产业体系、加快转变发展方式等一系列政策措施，积极推进“三促进一保持”，构建以服务经济为主体，现代服务业为主导，高新技术产业与先进制造业融合发展的现代产业体系，三次产业比例由“十五”期末的2.5︰39.7︰57.8调整为1.8︰37.2︰61。引进建设一大批高端产业项目，五年累计安排重大建设项目141个，投资总额达3401亿元，是“十五”时期的2.8倍。加强与中央企业及国内大型企业的战略合作，签约项目总投资额超3000亿元，有力促进了产业结构的优化升级。服务经济主体地位明显增强。2010年，全市第三产业增加值达6464.79亿元，同比增长13.2％，是2005年的1.96倍，对经济的贡献率达61.2％。区域金融中心建设取得新进展，2010年全市新增金融机构15家，总数达191家，比2005年增加71家，金融业增加值达615.54亿元，同比增长8.2％，是2005年的3.09倍。现代商贸业快速发展，2010年全市实现社会消费品零售总额4476.38亿元、商品销售总额21204.27亿元，分别比2005年增长1.3倍和1.9倍，获“福布斯2010年中国大陆最佳商业城市”排行榜第一名。广交会、广博会等品牌会展影响力不断扩大,现代物流业加快发展。文化及相关产业成为新的经济增长点，2010年实现增加值843.45亿元，同比增长13.6％。旅游体系建设和产品开发成效明显，2010年全市旅游接待人数达1.27亿人次，旅游总收入突破1000亿元，分别为2005年的1.35倍和2.01倍。汽车、造船、石化、精品钢、核电装备等先进制造业不断发展壮大，加速向资本密集、技术密集和人才密集转型升级。2010年，全市规模以上工业总产值达14721.47亿元，同比增长17.6％，是2005年的2.13倍；实现规模以上工业利润970.25亿元，是2005年的2.4倍。汽车产量达135.84万辆，实现规模以上汽车制造业产值2878.44亿元，分别比2005年增长2.3倍和2.2倍。特别是首款自主品牌乘用车“传祺”下线、30.8万吨超大型油轮下水和国内首台国产化百万千瓦级核电压力容器研制成功，标志着我市自主研发和工业制造能力实现新突破。自主创新能力不断增强，高新技术产业快速发展。预计2010年全市科技研发经费支出占GDP比重达2.4％，比2005年提高0.75个百分点。专利申请量和授权量分别突破2万件和1.5万件，比2005年分别增长88.8％和163.6％。全市规模以上高新技术产品产值占规模以上工业总产值比重达38.5％，超过“十一五”预期目标8.5个百分点，比2005年提高10.1个百分点。大力扶持民营经济加快发展。2010年，全市民营经济增加值4056.05亿元，同比增长13.5％。产业“退二进三”和“双转移”步伐加快。2008年以来，全市关停、搬迁企业（项目）5699家（个），向市外转移企业175家，淘汰落后产能1425万吨，在全省“双转移”工作考核中并列第一。节能减排工作扎实有效。“十一五”期间，预计万元生产总值能耗下降20.4％，规模以上单位工业增加值能耗下降39.3％，二氧化硫和化学需氧量排放总量分别从14.9万吨和13.7万吨下降到8.5万吨以下和11万吨以下，超额完成“十一五”规划的减排目标。

　　（二）举全市之力筹办亚运会和亚残运会。

　　自成功申办以来，我们认真贯彻中央和省的决策部署，精心筹划，严密组织，夜以继日，扎实工作。特别是2010年，筹办工作进入冲刺阶段，我们以一流的组织指挥、一流的场馆设施、一流的城市环境、一流的服务水平、一流的安保措施、一流的社会风尚，上下一心、攻坚克难、奋力冲刺，夺取了筹办工作的全面胜利。一是开闭幕式精彩绝伦。亚运会开闭幕式，以珠江为舞台，以城市为背景，创意无限，气势恢宏；亚残运会开闭幕式，突出“大爱感人、自强不息”的主题，充分展现了广州包容和谐的城市精神。二是赛事运行圆满顺利。亚运指挥部和各工作团队运转高效，各项赛程转换有序，确保了赛事顺利进行。三是服务保障优质高效。在迎送宾客、交通住宿、信息通信、电力供应、饮食安全、医疗卫生、宗教礼仪等各个方面都作了周到细致的安排，实现了安全生产零事故、食品安全零事件、礼宾接待零失误、接待服务零投诉、运行保障零疏漏。四是安保维稳万无一失。5万多名部队官兵、3万多名公安干警、近万名民兵预备役人员和80多万干部群众参与亚运安保维稳。整个赛会期间，全市社会治安秩序良好，实现了“平安亚运”的目标。五是社会氛围热烈祥和。充分发挥中央和省、市主流媒体的主导作用，营造了良好热烈的舆论氛围，宣传报道亮点突出，全方位、多角度展示了亚运风采。在全社会大力弘扬讲文明、重礼仪、团结友善、热情好客的良好风尚，特别是58.5万名亚运志愿者以“一起来、更精彩”的理念踊跃参与志愿服务，充分体现了广州人民空前高涨的主人翁精神。两个盛会的成功举办，创造了无愧于祖国、无愧于人民、无愧于时代的业绩，留下了丰富的物质遗产和宝贵的精神财富，对于进一步推进广州改革开放和现代化建设具有重大意义，必将彪炳史册。

　　（三）全面推进城市环境面貌实现“大变”。

　　一是强化城市规划的引领控制。按照“南拓、北优、东进、西联、中调”的城市发展战略，进一步完善城市总体规划和土地利用总体规划，引领城市建设和发展，优化区域协同发展格局，推进十区控规全覆盖，完善综合交通网络，保护城乡生态环境，强化旧城保护与更新，推进珠江新城、亚运新城、白云新城、萝岗新城等新城区建设，多中心、组团式、网络型城市空间布局进一步凸显。广州城市战略规划荣获国际杰出范例奖。二是加大基础设施建设力度。“十一五”时期,共投入2133亿元推进交通基础设施建设。白云国际机场二期，南沙港区二、三期工程加快推进，广州南站建成投入使用；新建177公里地铁线路、210公里高速公路和609公里城市道路。白云国际会议中心等一批标志性建筑相继建成，供水、供电、供气等市政公共设施不断完善。三是推进城市环境综合整治。以实现水环境、空气环境、人居环境、交通环境和无障碍环境“大变”为主要内容，开展了大规模城市环境综合整治。新铺设1094公里截污管网，建设污水处理厂38座，新增污水处理能力236.58万吨／日，全市生活污水集中处理率提高到85％，中心城区近90％。对121条河涌进行综合整治，建设白云湖、海珠湖，主要河涌和珠江水质持续好转。整治水浸街227处，城市排水系统进一步完善。铁腕治污，落实“空气整治50条”和“新31条”，重点治理工业企业等七大类污染源，公交车、出租车推广使用清洁能源，中心城区全面禁行摩托车，对黄标车实行限行措施。2010年空气质量优良率为97.81％，比2005年提高6.85个百分点，连续5年优于国家二级标准。着力改善人居环境，升级改造177条共483.63公里市政道路；整饰建筑31190栋共3600多万平方米；对160个社区实施雨污分流改造；推进绿化美化工程，建成区绿化覆盖率达40.15％。“三旧”改造步伐加快。大力改善交通环境，2010年，优化和新开通165条公交线路，建成22.9公里快速公交（BRT）试验段，交通拥堵专项整治工作取得初步成效。加快无障碍设施建设，新建和改造盲道1129.34公里、坡道11827个；在500个红绿灯路口安装盲人过街音响信号装置；投放961台无障碍公交、出租车辆；对321座环卫公厕进行无障碍改造。通过艰苦卓绝的努力，“大变”目标全面实现，城市环境面貌明显改善。四是加强城市管理。理顺市、区两级城市管理体制，下放管理权限，完善以区街为管理主体、多部门综合治理的城市管理联动机制，有效提高了管理水平。开展为期三年的“城市管理年”活动，尤其是结合迎亚运工作，严格执法，重拳治乱，依法拆除133万平方米违法建设，清理拆除户外违法广告招牌近5万块，有效遏制了“六乱”现象。大力整顿和规范市场经济秩序，食品药品安全体系进一步完善，五年来没有发生重特大食品药品安全事故。加强流动人员和出租屋管理，进一步完善社会治安防控体系，治安形势明显好转，2010年全市刑事案件发案数同比下降10.3％。安全生产形势保持稳定，2010年事故起数、死亡人数比2005年分别下降47.29％和41.75％。

　　（四）大力加强以改善民生为重点的社会建设。

　　“十一五”期间，市本级财政教育支出累计达107.8亿元，比“十五”时期增长135.49％。100％的区（县级市）和镇成为省教育强区、强镇，普通高考连续21年处于全省龙头地位，中等职业教育保持全国领先，高等教育位居全国同类城市先进行列。大力推进文化强市建设，成功举办第九届中国艺术节，广州塔等20多个重点文化设施建成投入使用。深入开展全国文明城市创建活动，城市文明程度进一步提升。体育事业蓬勃发展, 全市社区体育设施配置率达到95％以上，98％以上行政村建有标准篮球场,广州籍运动员在国内外重大比赛中取得好成绩。成功举办第八届全国少数民族传统体育运动会。建成我国内地首个“无规定马属动物疫病区”。圆满完成汶川威州镇灾后重建等对口支援任务，援藏援疆工作扎实推进，扶贫开发“双到”工作取得阶段性成果。大力加强国防动员建设，深入推进“双拥”和优抚安置工作，连续六次荣获全国、连续七次荣获全省“双拥模范城”称号。人口计生、民族宗教、妇女儿童、青少年、助残、侨务、对台、统计、参事、文史、修志、档案、气象、民防等各项事业都取得长足进步。五年来，我们始终把保障和改善民生摆在突出位置，出台“惠民66条措施”和“17条补充意见”，让广大市民群众共享改革发展成果。2010年，市本级财政投入民生和各项公共事业资金达317.26亿元，占一般预算支出的75％。2010年十件民生实事全部兑现：一是城镇新增就业人数97万人，超额完成新增就业20万人的目标。二是新增社会保险参保人数225万人次，超额完成年度扩面任务，企业退休人员养老金月人均达到2229元，农转居人员养老金月人均达到567元。三是再次提高城乡最低生活保障标准，城镇和农村平均低保标准分别达到398元和303元。四是市第八人民医院第一期、市疾控中心、市妇女儿童医疗中心建成投入使用，免费为18万名15岁以下儿童补种乙肝疫苗，为18.1万名农村妇女开展“两癌”检查。五是如期完成西江引水工程，直接惠及600万市民，全市饮用水水源地水质100％达标。六是基本完成新增100所义务教育规范化学校的任务，义务教育规范化学校比例达79.3％。七是新开工建设4万套300万平方米保障性住房，累计解决6.83万户低收入家庭住房困难问题，完成总目标的89％。八是新建和续建一批城市道路，新建6个公交枢纽站,更新1907辆老旧公交车，翻新改造6673辆出租车和4312辆公交车, 新增6.13万个停车泊位，市民群众出行条件进一步改善。九是建设500处社区健身路径等体育场地，建成1060公里的绿道，超额完成省下达的526公里建设任务。十是政府部门共有143项面向市民和企业的服务事项实现网上办理，超额完成既定的100项任务。

　　（五）加大重点领域和关键环节改革力度。

　　继续深化经济体制改革。推进国有企业改革向纵深发展，国有企业公司制改造基本完成，法人治理结构不断完善，发展活力不断增强，2010年市属经营性国有企业实现利润总额217.25亿元，同比增长54.5％。组建七大投资集团，在城市建设和环境整治中发挥了重要作用。市属金融机构改革重组取得历史性突破，广州银行和广州农村商业银行彻底解决了历史遗留问题，实现了跨区域经营。发展和利用资本市场取得新进展，2010年新增12家上市公司，全市上市公司累计达70家，是2005年的2.2倍。特别是国有企业上市步伐加快，21家国有控股上市公司市值比上年增加一倍，净增1100亿元。深入推进行政管理体制改革。全面完成新一轮政府机构改革，市政府工作部门和办事机构由49个精简为40个，进一步整合行政资源，优化组织结构，理顺职责关系。开展新一轮行政审批制度改革，行政审批事项由816项减少到275项，五年累计减轻群众及企业负担49.3亿元。启动事业单位岗位管理改革，全市3579家事业单位共14.5万人实现以岗位管理代替身份管理。学习借鉴香港先进经验，创新社区管理服务方式。文化体制改革逐步深入，经营性文化单位转制和大型文化企业集团重组取得突破。

　　（六）深入推进对外开放和区域合作。

　　对外经济贸易稳步发展。2010年，广州地区进出口总值1037.8亿美元，同比增长35.3％，是2005年的1.9倍；其中，出口总值483.8亿美元，同比增长29.3％，是2005年的1.8倍。实际利用外资40.81亿美元，同比增长5.3％，五年累计实际利用外资181.96亿美元，比“十五”时期增长43.6％。世界500强企业已有174家落户广州。加快推进区域一体化发展。广佛同城化83项重点合作项目和广佛肇经济圈建设37项主要工作顺利推进，国内首条全地下城际轨道交通广佛线建成通车，医疗、通信、金融、环保等区域公共服务一体化建设取得实质性进展，在省实施《规划纲要》珠三角九市考核中名列第一。加强与港澳台在经济贸易、现代服务业和节能环保等领域的交流合作，南沙保税港区和白云机场综合保税区获国务院批准设立，南沙开发区获省政府批准为实施CEPA先行先试综合示范区。组团参加“台湾·广东周”活动，进一步推动了穗台经贸文化交流与合作。积极拓展对外交流，国际友好城市和国际友好合作交流城市增至36个。我市行政首长成功连任世界城市和地方政府组织（UCLG）联合主席，大大提升了广州的国际知名度和全球影响力，体现了当今世界对广州的高度关注和认同，为我市进一步参与国际交流与合作，搭建了更加宽广的平台，“大外事”格局初步形成。

　　（七）大力促进城乡一体化发展。

　　坚持工业反哺农业、城市支持农村的方针，出台一系列强农惠农政策，促进农业农村发展。2010年市本级财政投入涉农资金59.05亿元，增长12.78％，五年累计投入248.98亿元，比“十五”时期增加1.3倍。加快推进城市基础设施向农村地区延伸，全市各级财政累计投入超过15亿元，全面完成1142个行政村“五通”工程。加强水利工程建设，城乡防御灾害能力显著增强。着力改善农民生产生活条件，推进1100个村的基础设施建设和村容村貌整治，建成296个农村生活污水处理设施，在68个村开展农村生态示范村创建工作。以中心镇建设为抓手，加快农村城镇化进程，五年累计投入55亿元，推进17个中心镇基础设施建设，中心镇人口和产业集聚功能明显增强。在全省率先开展统筹城乡综合配套改革试点，进行城乡一元化户籍制度改革和土地承包经营权流转试点。大力发展都市型现代农业，累计完成146万亩标准化农田和15万亩标准化鱼塘建设，蔬菜、畜禽、花卉、水产养殖等特色农业快速发展。加强农产品标准化和质量安全监管体系建设，大力实施科技兴农，扶持农民专业合作社和农业龙头企业发展，农业综合生产能力进一步提高。2010年全市农业总产值323.4亿元，同比增长3.5％。千方百计促进农民增收。五年累计培训本市农村劳动力30万人，转移就业32万人。壮大发展农村集体经济，扶持发展乡村旅游，拓展农民增收渠道。五年来，农民收入增幅有四年超过城镇居民，提前实现人均纯收入一万元的目标。

　　（八）大力加强民主法制和政府自身建设。

　　认真贯彻市人大及其常委会各项决议，定期向市人大、政协报告、通报工作，自觉接受法律监督、工作监督和民主监督。五年来，共办理人大代表建议1917件、政协提案2570件，办复率达100％。大力推进依法行政，五年共完成地方性法规草案23项，出台政府规章43项，修改和废止政府规章73项。2010年出台或提请省政府、市人大常委会制定了10部亚运保障相关法规和规章，为成功举办亚运会和亚残运会创造了良好的法制环境。市政府被国务院法制办确定为规范性文件管理示范点。科学化、法制化、民主化决策机制进一步完善。强化行政执法责任制，加大对领导干部问责力度。积极推进政务公开和电子政务建设，市政务服务中心公共服务质量不断提高。加强政府系统廉政建设，加强对亚运筹办工作监督检查和跟踪审计，亚运城及各项设施建设共节省资金10.3亿元，实现了“廉洁办亚运、节俭办亚运”的目标。大力精简会议、文件，机关作风明显转变，行政效能进一步提高。

　　各位代表，过去的五年，困难巨大，挑战巨大，付出巨大，收获巨大。成绩来之不易，经验弥足珍贵。我们深刻体会到，推动广州科学发展，一是必须注重抢抓机遇，增创优势，增强忧患意识、责任意识和风险意识，勇于先行先试，主动应对挑战，促进率先发展、和谐发展；二是必须注重转型发展，加快结构调整，落实“三促进一保持”，实现经济又好又快发展；三是必须注重科教引领、创新驱动，增强自主创新能力，促进创新发展；四是必须注重优化环境，加强生态建设，提升城市品质，实现经济社会发展与人口、资源、环境相协调，促进绿色发展；五是必须注重深化改革，扩大开放，坚持先行先试，加强制度创新，正确处理政府与市场的关系，着力增强城市发展活力，不断提升城市国际化水平；六是必须注重改善民生，富民惠民，创新社会管理和服务，实现发展成果由全民共享，不断提升市民群众的幸福感。

　　各位代表，过去五年取得的巨大成就，归功于党中央、国务院和省委、省政府及市委的正确领导，归功于市人大及其常委会和市政协的有力监督和支持，归功于全市人民的辛勤劳动。尤其在亚运筹办期间，全市人民充分理解，克服困难，全力支持，广泛参与，作出了重大贡献。在此，我谨代表广州市人民政府，向全市人民、驻穗解放军和武警部队官兵、各民主党派、人民团体以及社会各界人士表示崇高的敬意!向所有关心和支持广州现代化建设的港澳同胞、台湾同胞、海外侨胞和国际友人表示衷心的感谢！

　　在充分肯定成绩的同时，我们清醒地看到，我市经济社会发展仍然面临不少问题和困难，主要是：经济发展方式仍然比较粗放，产业层次不够高，特别是传统服务业比重大，自主创新能力还不强，研发投入长期低于京、沪、深等城市，研发产业化水平相对较低，产业核心竞争力还不够强，结构调整、转型升级任务艰巨；资源和环境约束突出，节能减排形势依然严峻；城市间竞争日趋激烈，建设国家中心城市面临诸多挑战；人口总量快速扩张给城市管理带来新情况、新考验，城市管理科学化、精细化水平亟待提升；城乡发展还不平衡，公共服务供给还不能满足市民群众日益增长的需求，就业、社保、教育、医疗、住房保障等民生工作有待加强；政府工作还存在不足，转变政府职能和加强作风建设仍须努力。我们必须正视存在问题，采取措施认真加以解决。

　　二、“十二五”时期奋斗目标和主要任务

　　“十二五”时期是广州国家中心城市建设全面提速，率先加快转型升级、建设幸福广州的关键时期。机遇与挑战并存，竞争与合作同在。我们必须认清形势，强化危机意识、忧患意识，勇于解放思想，勇于改革创新，勇于先行先试，以全球视野、战略思维、创新意识，把广州的发展置于国家中心城市的战略高度和世界先进城市的坐标体系中来谋划部署，抢抓机遇，应对挑战，奋发进取，扎实工作，努力开创广州科学发展的新局面。

　　“十二五”时期我市经济社会发展的指导思想是：高举中国特色社会主义伟大旗帜，以邓小平理论和“三个代表”重要思想为指导，深入贯彻落实科学发展观，全面实施《规划纲要》，坚持以科学发展为主题，以加快转变经济发展方式为主线，以全面建设国家中心城市为目标，以建设国际商贸中心和世界文化名城为战略重点，以深化改革开放为动力，以切实保障和改善民生为根本出发点和落脚点，充分发挥亚运后续积极效应，按照“科学发展、先行先试”的要求，率先加快转型升级、建设幸福广州，全面提升城市核心竞争力、文化软实力和国际影响力，推动现代化国际大都市建设迈向新阶段。

　　“十二五”时期经济社会发展的主要目标是：转变经济发展方式取得重大进展，综合经济实力继续保持全国领先，宜居城乡建设取得重大进步，改革开放进一步深化，社会建设全面加强，人民生活水平明显提高，群众幸福感明显增强，国家中心城市建设迈上新台阶。到2015年，全市生产总值达到1.8万亿元，年均增长11％左右;“十二五”期间，城市居民人均可支配收入和农村居民人均纯收入年均增长10％以上。重点在“三个重大突破”和“五个全面推进”上下功夫：

　　“三个重大突破”：一是战略性基础设施要有重大突破。重点建设国际航空枢纽、沿海主枢纽港、铁路主枢纽、城际轨道交通、地铁交通、公路主枢纽、能源保障和枢纽型国际化信息港等战略性基础设施，大幅提升国家中心城市建设的支撑力和承载力。二是战略性主导产业要有重大突破。整合各项扶持产业发展资金，力争市、区两级财政每年安排40亿元，5年共安排200亿元资金，凝聚力量，形成拳头，重点支持对我市产业高端发展起重要引领作用的金融保险、商贸会展、现代物流、文化创意、信息、汽车、石油化工、重大装备、生物和新材料等重大战略性主导产业，整体提升我市产业核心竞争力，增强发展后劲。三是战略性发展平台要有重大突破。按照东部建设高新技术产业带、南部建设高端装备制造业产业带、中部建设中央现代商务和文化产业带、北部建设生态经济产业带的产业发展空间布局，重点谋划建设中新广州知识城、南沙新区、三大国家级开发区、六大现代服务业功能区、空港经济区、天河智慧城和白云国际健康产业城等战略性发展平台。各区（县级市）也要谋划建设2至3个对全市产业发展有重大影响的载体，形成推动经济增长的新引擎。

　　“五个全面推进”：一是全面推进国际商贸中心建设。以现代服务业为主导的现代产业体系基本建立，营商环境与国际全面接轨，商贸会展、现代物流、金融服务、高端商务等功能进一步增强，初步建成具有较强全球辐射力的国际商贸中心。二是全面推进世界文化名城建设。公共文化体系建设和文化产业竞争力位居全国前列，教育现代化水平不断提高，城市文明程度和市民整体素质显著提升。岭南文化进一步传承发扬，对外文化交流合作日益活跃，初步建成开放、包容、多元的世界文化名城。三是全面推进国家创新型城市建设。创新资源加快集聚，创新主体活力明显增强，创新服务体系不断完善，成为国家战略性新兴产业重要基地和创新型国家的重要战略支撑；改革创新取得重大进展，与国家中心城市科学发展相适应的体制机制基本形成。四是全面推进综合性门户城市建设。辐射全国、连通世界的现代化枢纽型基础设施体系更加完善，国际航运中心功能进一步强化；城市国际化程度明显提高，建成我国南方对外政治、经济、文化交流的核心门户。五是全面推进全省宜居城乡“首善之区”建设。城乡规划建设管理水平显著提升，生态环境质量明显改善，花园城市基本建成。就业和社会保障水平稳步提高，社会管理、城市管理更加人性化、精细化，社会更加和谐稳定，城乡居民收入较快增长，城乡一体化发展格局基本形成。

　　三、2011年工作安排

　　今年是实施“十二五”规划的开局之年。我们要按照市委九届十次全会关于今年工作的指导思想和总体部署，准确把握科学发展的主题，准确把握转变经济发展方式的主线，把亚运创造的巨大精神财富转化为加快发展的强大动力，进一步凝聚全市人民的智慧和力量，以更加饱满的热情和更加昂扬的斗志，推动经济社会发展再上新台阶，为实现“十二五”规划目标任务开好局、起好步。

　　经济社会发展的主要预期目标是：地区生产总值增长11％；人均生产总值增长10％；全社会固定资产投资增长10％；社会消费品零售总额增长16％；外贸出口总值增长10％；万元生产总值能耗下降3％以上；二氧化硫和化学需氧量排放量分别下降1％和2.5％；城市居民人均可支配收入增长11％；农村居民人均纯收入增长12％；居民消费价格总水平涨幅控制在4％左右；城镇登记失业率控制在3.5％以内；人口自然增长率控制在6.2％。以内。

　　实现上述目标任务，必须突出“六个重点”，即突出转型升级，突出自主创新，突出惠民优先，突出城乡统筹，突出改革创新，突出开放合作；切实抓好以下“八项重要工作”:

　　（一）促进内外需协调发展，确保经济实力位居全国城市前列。

　　强化重点项目带动作用。以优化结构、提高效益为重点，大力推进101个重点项目建设，特别要发挥60个总投资20亿元以上特大项目建设的带动作用，力争全年重点项目投资826亿元，带动全社会投资约3600亿元。进一步调动和发挥民营企业投资积极性，抓好100项民营投资项目建设，鼓励和引导民间资本参与城市基础设施、现代产业和公用事业等领域的投资。加强重点项目管理，确保项目早建成、早投产、早见效。

　　加大招商引资力度。按照大项目带动大发展的思路，切实做好重大项目的引进、储备工作。积极开展面向大型央企、全国500强民企及世界500强企业的招商引资活动。组织“新广州、新商机”主题招商推介系列活动，策划科技专项招商。优先引进资金投入大、技术含量高、产业链条长、带动能力强、符合我市产业发展方向的战略性主导产业项目，重点吸引跨国公司、中央企业、行业龙头企业尤其是总部企业投资落户，形成“建成投产一批、开工建设一批、谋划储备一批”的良性发展机制，切实增强发展后劲。

　　增强消费拉动力。鼓励多种形式就业创业，多渠道增加城乡居民收入，提升居民消费能力。着力建设白云新城等一批具有国际水平的高端商贸集聚区，构建“社区商业10分钟便利消费圈”，不断改善和优化消费环境。建设和改造提升广州美食园等一批美食集聚区，擦亮“食在广州”金字招牌。组织开展“广货北上、西行”和“家电下乡”等活动，开拓城乡消费市场。积极开发后亚运旅游产品，推进北京路广府文化和西关商贸文化旅游产业带建设，打造旅游新品牌，吸引更多国内外游客到广州旅游消费。

　　积极利用和发展资本市场。抢抓机遇，整合资源，推进区域金融中心建设。发挥资本市场的放大功能和裂变效应，促进经济加快发展。构建企业上市“绿色通道”，推进企业上市“双百工程”，力争今年新增15家企业上市。拓宽企业融资渠道，加快发展债券市场，大力发展股权投资市场，争取广州高新区列入国家代办股份转让系统扩大试点园区。加快广州产权交易市场发展，推进广州交易所集团国际合作进程，积极创造条件，争取恢复设立广州期货交易所。

　　保持进出口平衡和利用外资持续增长。创新对外贸易发展方式，全力巩固传统市场，稳步开拓新兴市场。进一步优化出口产品结构，鼓励具有自主品牌和高附加值产品增加出口。加快培育本土跨国企业，鼓励有条件的企业“走出去”，拓展外贸市场，参与境外投资。积极推动加工贸易转型升级，大力发展服务贸易，提升服务外包发展水平。推进南沙保税港区和白云机场综合保税区建设，全面提升经济国际化水平。

　　（二）以发展壮大核心产业为突破口，推动经济转型升级。

　　做大做强核心产业。扬长避短，发挥优势，打造核心企业，培育核心产品，提升产业核心竞争力。实施“百强工程”，集中优势资源，扶持具有行业比较优势的100项重大产业项目发展。大力发展现代服务业，着力发展金融总部经济，培育大型商贸龙头企业，提升会展业发展水平，促进物流业快速发展。加快发展自主品牌汽车，打造高端机床、轨道交通、船舶制造、核电装备及输变电设备等国家重大成套和技术装备基地。全力推进8.5代液晶平板显示器、高亮度大功率发光芯片和新岸线CPU芯片产业化基地、华南新药创制中心、工业胶新材料生产基地等重大产业项目建设。力争经过3至5年的努力，形成一批特色鲜明、体系完整、环境友好、品牌响亮、有强大国际竞争力的核心产业集群。

　　培育发展战略性新兴产业。市本级财政安排20亿元资金用于扶持战略性新兴产业，提升产业核心竞争力，增强可持续发展能力。大力发展新一代信息技术、生物工程技术、新材料、新能源汽车、新能源与节能环保、海洋产业等战略性新兴产业，打造国家级战略性新兴产业基地，抢占经济与科技制高点。把握我市列入国家节能与新能源汽车示范城市和广东启动国家低碳省试点工作的契机，加快发展纯电动和混合动力汽车，加快发展核电、风电、太阳能、生物质能及半导体照明、环保装备等新能源与节能环保产业，形成LED半导体照明产业集群。推进中国软件名城创建工作。积极申报国家海洋经济综合开发示范区。

　　壮大发展民营经济。从今年开始，连续5年每年安排财政资金1亿元，奖励作出重要贡献的民营企业。实施“民营企业上市培育工程”和“中小企业成长工程”，着力解决民营企业融资难问题。出台民营投资产业导向目录，降低民间资本准入门槛。支持广州民营科技园扩园发展，加快建设成为国家民营科技示范基地。抓好专业镇产业转型升级。加快培育一批进入全省百强民企行列的大型企业，促进民营经济发展壮大。

　　加快“双转移”和产业“退二进三”步伐。抓住“三旧”改造的有利政策和时机，加大产业和劳动力“双转移”力度，启动第二、第三批“退二”企业搬迁工作。加快淘汰落后产能，引导、规范和提升“五小企业”发展，为高端产业发展腾出空间。

　　抓好节能减排工作。大力发展循环经济，加快能源结构调整，提高资源综合利用效率。加快环卫设施升级改造和生活垃圾分类收集工作。推进建设李坑生活垃圾焚烧发电二厂、固体再生资源中心等项目，实现工业固体废物、电子废弃物和生活垃圾的减量化、资源化和无害化处理。在政府机关带头推行低碳绿色办公，开展低碳宣传教育，引导市民群众选择低碳生活方式和绿色消费，建设低碳广州。

　　（三）充分发挥创新资源集中的优势，加快建设国家创新型城市。

　　完善自主创新体系。编制实施建设国家创新型城市行动计划，加快建立技术创新体系，依托高校和科研机构的资源力量，搭建公共技术平台，开展核心和关键技术创新研究，推动经济发展方式转入创新驱动轨道。发挥财政资金引导作用，不断提高科技研发投入。加强科技中介服务体系建设，促进科技创新成果转化。实施知识产权战略规划，大力加强专利、商标、版权等自主知识产权培育创新工作，加快专利技术产业化步伐，形成一批拥有自主知识产权、具有较强竞争力和重大影响力的企业品牌。积极支持优质企业推进国际质量认证、环境管理体系认证和行业认证，支持企业推出技术标准。

　　强化企业创新主体地位。实施创新型企业示范工程，促进各类创新资源向企业集聚。鼓励大型企业加大研发投入，激发中小企业创新活力，培育一批带动能力强的创新型企业，形成以创新型企业为龙头的高新技术产业集群。支持大中型企业建立研发机构，鼓励有条件的企业扩大国际交流合作，提高企业技术创新能力。

　　构筑国际人才高地。实施“万名海外人才集聚工程”和“创新创业领军人才百人计划”，充分发挥和提升“留交会”等平台作用，完善和落实有关优惠政策，不断优化人才发展环境，大力培养和引进科技领军人才、创新创业人才、创新团队和高技能人才，为广州发展提供强有力的人才保障。

　　（四）大力加强城市规划、建设和管理，不断提升国家中心城市功能。

　　优化提升城市规划水平。加快完成《广州市城市总体规划（2010－2020）》编制工作，按照建设国家中心城市的目标定位，进一步优化城市空间布局，推动城市空间布局由外延拓展向内涵优化提升转变，加快形成人口、产业、资源和环境相协调的城市空间格局。重点打造“一条中轴线、两个新城区、三个片区中心”。城市新中轴线以建成高品质城市核心带为目标，加快商务金融、商贸会展、行政办公、旅游观光等现代综合服务高端要素的集聚。东部新城依托中新广州知识城、科学城和增城开发区，打造山水新城，成为广州东部重要的创新中心和综合性核心城区。南沙新城以南沙港为依托，建设成为宜居宜业的现代化滨海新城。花都新华、增城荔城、从化街口要率先建成统筹城乡发展的现代化新城区。

　　推进城市基础设施建设。抓紧建设白云国际机场第三跑道、第二航站楼及综合换乘枢纽，构筑功能完备的航空物流中心和空港商务中心。加快广州港出海航道三期、南沙粮食及通用码头工程、南沙集装箱码头三期和南沙疏港铁路、黄埔疏港道路体系等港口基础设施建设，提升港口功能。积极支持配合国家和省加快推进贵广、南广、广深港等高速铁路建设，加快启动穗莞深和佛莞城际线广州段建设，推进地铁六、七、九号线和广从轻轨项目建设。完成广河高速公路广州段、广深沿江高速广州段建设。切实抓好公共消防设施建设。

　　巩固城市环境综合整治成果。按照“巩固、完善、优化、提升”的原则，推进后亚运城市环境综合整治。加强涉水环境和水务设施管理，完善污水处理系统，提高污水处理率。推进珠江综合整治，实施河涌生态修复工程，努力建设与城市可持续发展相适应的洁净水体系。继续开展空气环境治理，推广清洁生产，深化降氮脱硝工作，推动企业实施集中供热或改燃清洁能源。强化机动车污染防治，全面实施国Ⅳ标准，加快淘汰黄标车。实施“宁静工程”，狠抓道路交通、建筑施工和饮食服务业等噪声监管。积极争取国家和省的支持，协调周边城市建立区域大气环境、水环境污染联防联治长效机制。巩固和深化“立面整饰”、“三线下地”、“雨污分流”工作，提升人居环境综合整治水平。继续推进“青山绿地”工程，提升园林绿化水平，建设“花园城市”。建立健全生态补偿机制，建设城乡一体的森林绿地体系。坚持节约集约用地，合理控制新增建设用地规模，严厉查处违法用地行为。继续推进“三旧”改造，今年新增改造面积不少于10平方公里。

　　加强城市科学化、精细化、常态化管理。着眼建立后亚运城市管理长效机制，进一步完善“两级政府、三级管理、四级网络”的城市管理格局，健全城市管理应急快速反应体系，规范城管综合执法行为，推动城市管理由治脏、治乱、治差向做细、做优、做靓转变，逐步建立起科学化、精细化、常态化的城市管理体系。加大违法建设查处力度。严厉打击“泥头车”无证运输、洒漏污染等行为。规范市场经济秩序，强化食品药品安全监管。加强出租屋管理，规范流动人员管理服务。抓好社会治安综合治理，完善社会面治安防控体系，严厉打击各类违法犯罪活动。强化安全生产工作，坚决遏制重特大安全事故。提升城市信息化应用水平。加快城市信息基础设施建设，进一步完善公共信息服务体系，促进经济社会运行管理和服务数字化、智能化。着力推进新一代通信网络、光纤入户、三网融合和物联网信息技术应用。启动超级计算机中心项目建设，实施“天云计划”，建设国际云计算中心平台，推进云计算技术应用和产业化。推进“无线城市”建设，在市政务服务中心、部分市级机关对外办事大厅，以及部分社区、商业网点、公园、旅游景点、图书馆、人才市场、医院候诊大厅、白云机场候机楼等公共场所和大中专院校，为市民群众提供免费无线宽带网络服务。扩大政府服务事项网上办理范围，提升政府在线服务水平。推进实施智能交通、智能电网、智能城管、智能家居以及天河智慧城、南沙智慧岛、黄埔智慧港等示范试点工程，加快建设智慧广州。

　　（五）继续推进先行先试，增强发展的活力和动力。

　　深化行政管理体制改革。加快推进以富县强镇、简政强镇和简政强区为主要内容的事权改革，推动管理重心下移，充分发挥区（县级市）的积极作用。深化大部门制改革，推进事业单位分类改革。抓好广州开发区创新发展模式、增城统筹城乡综合配套改革等改革试点工作。

　　加大国有企业改革力度。继续推进国有企业股份制改革，加快国有企业上市步伐，力争国有控股上市公司市值增长30％以上。完善法人治理结构，规范董事会建设，完善企业领导人选拔任用管理体制，建立健全长效激励和绩效考评机制。创新国有资产监管方式。推动国有资本加快向重点行业、关键领域、高端环节和优势企业集中，鼓励优势企业开展跨地区、跨行业和跨所有制重组，加快培育一批销售收入超500亿元的大型企业集团，推动我市国有经济布局优化和产业结构升级。

　　改革社区管理服务体制。与国家行政学院合作开展创新社会管理和文化管理重大课题研究。在部分街道开展社区管理服务体制改革试点，加强和改善社区管理服务。整合街道机构人员，优化基层管理结构，提高公共服务能力。实施社区"五个一"工程建设，为社区居民提供及时、优质的服务。建立和完善政府向社会组织购买公共服务制度。规范发展社会组织，加强社会工作人才队伍建设，构建政府与社会组织功能互补、相互协调的社会管理新格局。

　　提升区域合作水平。加快推进南沙实施CEPA先行先试综合示范区建设，积极拓展与港澳台在经贸、科教、文化等领域的交流与合作。认真贯彻实施《规划纲要》四年大发展工作方案，继续推进广佛同城化和广佛肇经济圈建设，统筹做好交通、水利、信息、能源保障等基础设施建设，开展重点协调地区同城化综合试验。加快提升环境保护、产业转移和产业协作水平，努力在珠三角一体化发展中发挥引领作用。继续做好援疆援藏等对口支援工作。

　　拓展国际交流的广度和深度。充分发挥我市作为UCLG联合主席城市的优势，积极利用其会员网络庞大、覆盖广泛的全球平台，加强与世界城市间的交往合作，争取与更多国际城市发展友好关系，不断提升广州的国际化水平和国际知名度。

　　（六）大力统筹城乡发展，进一步提升农村发展水平。

　　加快城市基础设施向农村延伸。统筹城乡资源配置，加快建设农村安居、污水治理、路网体系、供电网络、生活垃圾收集处理系统等重大项目，逐步形成覆盖到村、延伸到户、城乡衔接的基础设施网络。打造一批名镇名村。继续改善农村人居环境，开展以“两规、三清、四有、五通”为主要内容的村庄卫生保洁和周边环境综合整治，促进新农村建设。

　　大力发展农村公共事业。加大公共财政对农村的投入力度，加强农村社会保障，提高农村低保水平，推进农村养老保险全覆盖。完善农村基本医疗卫生制度，初步实现农村人人享有基本医疗卫生服务。加强农村文化基础设施建设，为农民提供更为丰富的文化产品。推动城区优质公共资源向镇、村延伸，促进城乡公共服务协调发展。

　　扶持发展都市型现代农业。加强农田水利基础设施建设，大力发展设施农业。继续推进农业经营组织化、规模化、产业化，加快和规范发展农民专业合作社。加快建设标准化、规模化、专业化生产基地，积极争创国家级或省级现代农业园区。完成集体林权制度改革。整合提升大型农产品批发市场，发展农产品配送和连锁经营业务。健全农产品质量保障体系，推进农业标准化生产。抓好“菜篮子”工程，建设万亩蔬菜设施栽培基地。大力推进农业科技进步，提高农业现代化水平。

　　拓宽农民增收渠道。加强农民就业培训，增强农民就业创业能力。加大农民外出务工就业指导和服务，新增农村劳动力转移就业5万人。壮大农村经济，促进农村集体经济结构调整和产业升级。大力发展农村地区服务业，加快发展生态旅游和观光休闲农业，拓展农民增收空间，不断提高农民收入水平。

　　（七）促进社会事业新发展，创造幸福美好新生活。

　　提升文化软实力。深入开展“讲文明树新风”、“做文明有礼广州人”等公民道德教育活动。大力弘扬亚运志愿者奉献精神，创新发展志愿服务事业。继续抓好文明社区、文明村镇、文明单位和文明家庭创建活动，整体提升城市文明水平和市民素质，创建全国文明城市。加快城市文化设施建设，深入挖掘和整合历史文化资源，开发和利用好亚运文化遗产，加强非物质文化遗产保护。落实“文化惠民”工程，打造公共文化服务体系国家级示范区和全国社区群众文化活动先行点。继续深化文化体制改革，加快发展文化产业，抓好群众性基层文化阵地建设，擦亮广府文化品牌，打造文化精品，繁荣文化事业。

　　加强各项社会事业建设。实行“五个压缩、一个增加”，即：压缩党政机关办公经费、公务接待经费、公务购车和用车经费、会议经费、出国经费，增加对民生和社会事业的投入。切实做好充分就业工作，全市新增就业30万人以上，其中本市生源毕业生就业率达到91％。加大对就业困难群体和“零就业家庭”的就业援助。健全完善农民工管理服务体系，加强人文关怀，保障劳动者合法权益，构建和谐劳动关系。继续扩大社会保险覆盖面，逐步解决我市农转居人员养老保险制度与城镇企业职工基本养老保险制度、新农保制度的并轨衔接问题。进一步推进医疗保险市级统筹，扩大基本医疗保障覆盖面，提高参保人医疗保障待遇。加快发展学前教育和特殊教育，继续推进义务教育规范化学校建设，提高普通高中教育办学质量，大力发展职业教育，提高市属高校办学水平。创建国家教育综合改革示范区。深化医药卫生体制改革，建立覆盖城乡居民的基本医疗卫生制度。大力推进镇卫生院、村卫生站和城市社区卫生服务机构标准化建设，加快基层卫生服务人才培养，完善社区和农村医疗卫生服务网络。全面实施基本公共卫生服务项目，完善基层医疗卫生机构补偿机制和运行机制。大力发展体育事业，倡导健康阳光的生活方式，加强体育人才培养，提升竞技体育综合竞争力。培育大型品牌体育赛事，统筹亚运场馆赛后利用，发展壮大体育产业。加强市场引导和监管，稳定消费价格总水平。加强和改进信访工作，畅通群众利益诉求表达渠道，认真做好矛盾纠纷排查化解工作，切实维护社会和谐稳定。加强计划生育服务管理，推动计划生育奖励政策城乡一体化，稳定低生育水平。发展老龄事业，建立健全养老社会服务体系。完善宗教事务管理，促进民族团结进步。做好对台和侨务工作，维护台胞和侨胞合法权益。加强防灾减灾基础设施建设，提高气象、地质、防震减灾工作水平。认真做好“规划到户、责任到人”扶贫开发工作，推进各项扶贫措施落实到位。开展“六五”普法工作，做好法律服务和援助工作。充分发挥工会、共青团、妇联等人民团体和社会组织参与社会管理和公共服务的重要作用。加强国防教育，推进“双拥模范城”建设。全面落实优抚政策，妥善安置转业、退伍军人和移交地方的军队离退休干部。加强民兵、预备役部队建设和海防、民防及打击走私工作。认真做好统计、档案、保密、参事、文史、修志等工作。

　　办好十件民生实事。一是做好扶贫开发工作。以我市北部山区8个贫困镇及其206个贫困村为重点，对全市379个贫困村开展对口帮扶，确保到2012年底，所有被帮扶的贫困村年集体收入基本达到10万元以上，被帮扶的贫困户家庭人均年收入基本达到5000元以上。二是免收贫困家庭学生中等职业教育学费。从2011年秋季起，对定为贫困家庭的本市户籍学生就读中等职业学校实行学费全免。三是继续扩大社保覆盖面。五大险种参保人数比上年增加82万人次，增长3.94％。其中新农保提前一年完成省下达的三年全覆盖任务，35岁以上农村居民全部纳入社保体系。四是提高城乡医疗保障水平。对政府举办的基层医疗卫生机构全面实施国家基本药物制度；提高城镇居民基本医疗保险、新型农村合作医疗财政补助标准，将新农合筹资标准从年人均260元提高到300元；进一步提高基本医疗保险参保人待遇，切实减轻市民群众看病就医的负担。五是提高社会救助水平。城乡居民低保标准提高17％至25％；资助8万名农村困难群众和重度残疾人参加新农保；孤儿养育标准提高至每人每月1000元。六是加大住房保障力度。构建以公共租赁住房为主的多层次住房保障供应体系。继续推进在建的6.87万套保障性住房建设，新开工建设4.3万套保障性住房，年底前全部解决登记在册的7.72万户低收入家庭住房困难问题。七是改善市民群众出行条件。新建、改造23条约81公里城市道路，新建人行过街设施27座。实施亚运后缓解交通拥堵方案，编制智能交通规划；大力发展公共交通，增设约70公里公交专用道，建设和改造24个公交站场，完善公交、地铁及其他运输方式的接驳，有效缓解交通拥堵问题。八是提高社会养老服务水平。将长寿保健金发放范围扩大到本市户籍70岁以上老年人；提高本市户籍80岁以上老年人长寿保健金标准，并为其免费安装“平安通”紧急呼援服务系统。九是推进农村道路“亮化”工程。建设5万盏农村路灯，使全市30％的农村道路实现“亮化”。十是为市民群众提供更多的休闲健身场所。新建240处社区健身路径、188个社区篮球场和一批全民健身小广场；新建500公里绿道，形成10条绿道休闲旅游精品路线。

　　（八）提高政府公信力和执行力，建设人民满意政府。

　　全面推进依法行政。坚决执行市人大及其常委会决议，切实做好人大代表建议、批评和意见及政协提案办理工作。坚持重大事项报告制度，自觉接受人大、政协和社会公众、新闻舆论监督。加强法治政府建设，做好后亚运城市管理立法工作。严格按照法定权限和程序行使权力、履行职责，规范行政许可自由裁量权等行政执法行为，不断提高科学决策和依法行政水平。发挥政府法律顾问和公职律师的作用。深入推行政务公开，推进网络问政常态化、制度化。健全完善行政问责制，强化政府公信力和执行力。

　　切实提高行政效能。规范行政审批行为，优化审批流程，推进跨部门并联审批，推动行政审批透明、规范、高效。大力弘扬亚运精神，增强全体公务员的服务意识、责任意识、奉献意识和进取意识。努力建设学习型政府，全面提升政府工作人员综合素质和服务水平。

　　加强政府廉政建设。落实党风廉政建设责任制，狠抓廉政风险防范管理。坚持廉洁办各项事业，建设节约型政府。充分发挥监察、审计等部门的监督职能，认真总结"廉洁办亚运"经验，开展对亚运资产处置工作的监督检查，加强财政资金监管和审计，努力从源头上防治腐败。切实纠正部门和行业的不正之风，着力解决损害群众利益的突出问题。

　　各位代表：回首过去，我们豪情满怀；展望未来，我们信心百倍。让我们高举中国特色社会主义伟大旗帜，坚持以邓小平理论和“三个代表”重要思想为指导，全面落实科学发展观，在市委的领导下，继往开来，奋勇前进，为率先加快转型升级、建设幸福广州而努力奋斗！
